# Gray matter alterations in restless legs syndrome: A coordinate-based meta-analysis

**DOI:** 10.1097/MD.0000000000021374

**Published:** 2020-07-17

**Authors:** LiQin Sheng, PanWen Zhao, HaiRong Ma, Liang Qi, ZhongQuan Yi, YuanYuan Shi, JianGuo Zhong, HaiCun Shi, ZhenYu Dai, PingLei Pan

**Affiliations:** a Department of Neurology, Kunshan Hospital of Traditional Chinese Medicine, Kunshan; b Department of Central Laboratory; c The Affiliated Huai’an Hospital of Xuzhou Medical University, Second People's Hospital of Huai’an City, Huai’an; d Department of Neurology; e Department of Radiology, Affiliated Yancheng Hospital, School of Medicine, Southeast University, Yancheng, P.R. China.

**Keywords:** coordinate-based meta-analysis, gray matter, restless leg syndrome, seed-based *d* mapping, voxel-based morphometry

## Abstract

**Background:**

Voxel-based morphometry (VBM) is an objective structural magnetic resonance imaging (MRI) technique which allows researchers to investigate group-level differences in regional gray matter (GM) volume or density over the whole brain. In the last decade, VBM studies in restless leg syndrome (RLS) have exhibited inconsistent and conflicting findings.

**Methods:**

Studies will be identified through a computerized literature search of the following databases: PubMed, Web of Science, and Embase until October 1, 2018 and updated on March 1, 2020. This protocol will be performed in accordance with the Preferred Reporting Items for Systematic review and Meta-Analysis Protocols (PRISMA-P). In addition, we will follow the recent guidelines and recommendations for coordinate-based meta-analysis (CBMA). This CBMA will be performed with the seed-based *d* mapping with permutation of subject images (SDM-PSI) software.

**Results:**

This CBMA will offer the latest evidence of GM alterations in RLS.

**Conclusions:**

To our knowledge, this will be the first CBMA that pooled VBM findings in RLS. This quantitative evidence of GM alterations will characterize brain morphometry of RLS.

**PROSPERO registration number:**

CRD42018117014.

## 1 Introduction

Restless leg syndrome (RLS) is a common sensorimotor disorder characterized by a distressing urge to move the legs due to unpleasant sensations, usually occurring or worsening during rest or at bedtime.^[1,2]^ RLS is of major clinical and public health significance due to its high prevalence, adverse effect on sleep and health-related quality of life, and a significant personal and social burden due to its increased risk of significant morbidity.^[2–5]^ Although the exact pathogenesis of RLS remains to be elucidated, brain dopaminergic dysfunction and iron deficiency play critical roles.^[6]^

Voxel-based morphometry (VBM) is an objective structural magnetic resonance imaging (MRI) technique which allows researchers to investigate group-level differences in regional gray matter (GM) volume or density over the whole brain. In the last decade, VBM studies in RLS have exhibited inconsistent and conflicting findings. The discrepancies in the VBM studies might be attributed to the small sample sizes in each single study, differences in methodological protocols (from magnetic MRI acquisition to statistics), and/or heterogeneity of RLS populations. Prior qualitative reviews therefore proposed that there might be no GM alterations in RLS.^[7]^ However, these VBM findings have not been quantitatively analyzed yet.

Coordinate-based meta-analysis (CBMA) is a powerful and invaluable approach to quantify voxel-based neuroimaging findings. In this study, we will use seed-based *d* mapping with permutation of subject images (SDM-PSI), to identify consistent and robust GM alterations in RLS.

## 2 Methods

### 2.1 Protocol and registration

This protocol will be performed in accordance with the Preferred Reporting Items for Systematic review and Meta-Analysis Protocols (PRISMA-P).^[8]^ The protocol of this meta-analysis was registered at PROSPERO (http://www.crd.york.ac.uk/PROSPERO) (registration number: CRD42018117014).

### 2.2 Data sources and study selection

Studies will be identified through a computerized literature search of the following databases: PubMed, Web of Science, and Embase until October 1, 2018. The search keywords used were (“voxel-based morphometry” OR “vbm” OR “gray matter” OR “grey matter” OR “voxel∗”) AND (“Willis Ekbom Disease” OR “restless legs syndrome”). No restriction to the publication language was used for the search. Additional qualified articles were obtained from the reference lists of relevant studies and reviews. The final search was updated on March 1, 2020.

### 2.3 Eligibility criteria

Studies will be included if they: enrolled patients with idiopathic RLS patients according to the accepted criteria and matched healthy controls (HCs); employed VBM analysis to investigate GM volume or density differences between RLS patients and HCs; reported significant imaging results with 3-dimensional coordinates either in Montreal Neurological Institute (MNI) or Talairach stereotactic space or null findings; were published as peer-reviewed and original articles in English.

**Figure 1:**
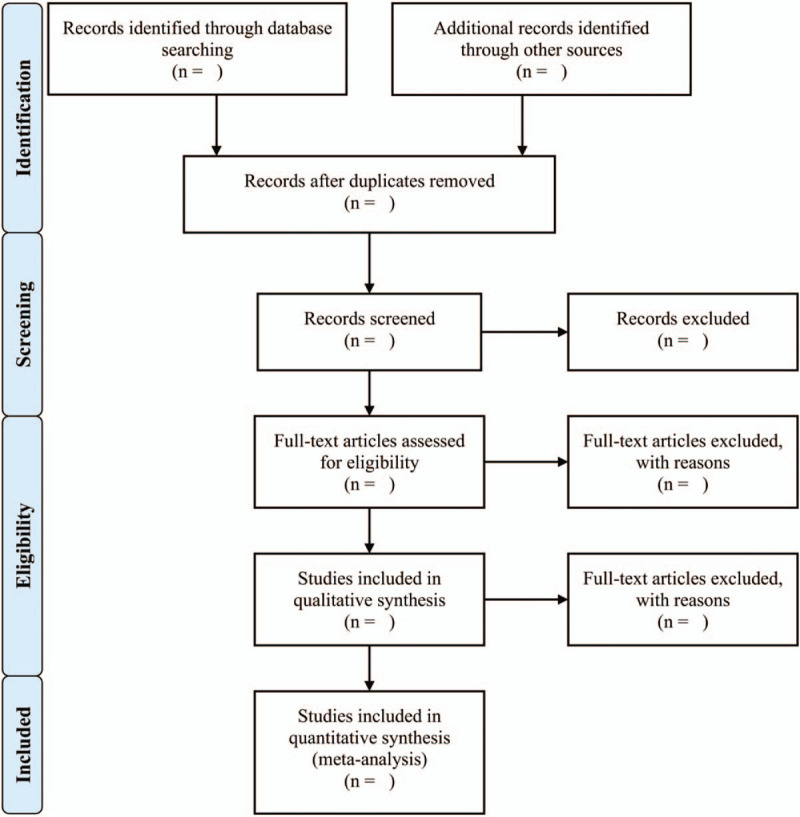
Study selection process in accordance with the PRISMA flowchart. PRISMA = Preferred Reporting Items of Systematic Review and Meta-Analysis.

Studies will be excluded if: the sample size in either the RLS group or the HC group was fewer than 7 individuals^[9]^; peak coordinates of significant results could not be obtained from the published articles even the authors had been contacted; region of interest (ROI) analysis or small volume correction (SVC) analysis was applied; when the patient group was overlapped in multiple studies, only the study with the largest sample size was selected; publications were not original articles, such as conference abstracts, letters, case reports, research protocols, reviews, and editorials. Figure 1 presents the study selection process in accordance with the PRISMA flowchart.

### 2.4 Data collection and extraction

For each included study, we will extract the following variables: name of the first author, publication year, sample size, age, sex distribution, International Restless Legs Syndrome Study Group (IRLSSG) severity scale (IRLS) score, illness duration, MRI field strength, MRI sequence, voxel size, imaging processing software package, template, modulation, processing methods, modulation, smooth kernel, covariate, statistical threshold, peak coordinates (*x*, *y*, and *z*), corresponding *t* statistics (*z* value or *P* value), and their stereotactic reference space, were extracted according to a predefined and standardized data extraction form.

### 2.5 Study quality assessment

Study quality of each study included will be assessed with a 10-point checklist based on previous neuroimaging CBMA.^[10]^ This checklist assesses aspects of clinical and demographic characteristics and imaging-specific methodology used in the studies (details in Table 1).

**Table 1:**
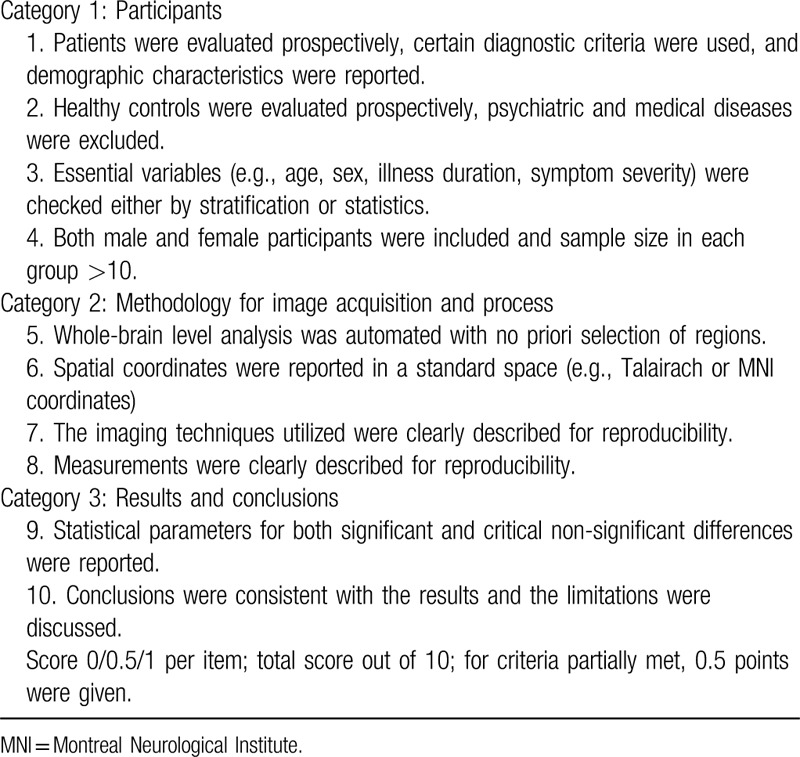
Quality evaluation checklists.

### 2.6 Data analysis

#### 2.6.1 Voxel-wise CBMA

This CBMA will be performed with the SDM-PSI software (www.sdmproject.com). SDM-PSI has been described in detail elsewhere.^[11,12]^ We briefly summarized the standard processes here. First, we collected and organized the information regarding the peak coordinates of significant GM differences between RLS and HCs. Second, the lower and upper bounds of possible effect size images were estimated within a GM mask. Third, effect sizes were analyzed using MetaNSUE based on multiple imputations algorithms.^[13]^ Fourth, Rubin rules are used to voxel wisely combine the meta-analysis images from the different imputed datasets.^[13]^ Finally, subject images were recreated in order to run a standard permutation test and the maximum statistic of the combined meta-analysis image is saved that the distribution of the maximum statistic is used to family-wise error-correct for multiple comparisons. The statistical threshold for this analysis was set to a corrected *P* < .05 (threshold-free cluster enhancement [TFCE]-based familywise error rate [FWER]) and voxels extent ≥10.

#### 2.6.2 Sensitivity analysis

To test the reliability of the results, sensitivity analysis will be performed by iteratively repeating the analysis leaving out 1 dataset each time.^[14,15]^

#### 2.6.3 Assessment of heterogeneity and potential publication bias

If there were significant results regarding consistent GM differences between RLS and HCs in the CBMA, we extracted the values from relevant peaks using PSI-SDM. Heterogeneity between studies was assessed with the *I*^2^ statistic using a random effects model. An *I*^2^ > 50% were regarded as indicators of heterogeneity. In addition, we applied funnel plots and Egger tests to assess the publication bias. An asymmetric plot and *P*-values <.05 were considered significant.

#### 2.6.4 Meta-regression analysis

Meta-regression analysis will be carried out to examine the effects of potential confounds, such age, female percentage in the sample, IRLS score, and illness duration on GM alterations across studies if these variables were reported in >10 datasets. The statistical threshold for this analysis was set to a *P* < .05 (TFCE-based FWER corrected) and voxels extent ≥10.

#### 2.6.5 Ethical principles and publication

No ethical approval is required because this coordinate-based meta-analysis will be performed based on published studies. The results of this review will be published in peer-reviewed journals.

## 3 Discussion

There is a debate regarding GM alterations in RLS. To our knowledge, this is the first CBMA that pooled VBM findings in RLS. Our CBMA will offer the quantitative evidence of GM alterations in RLS. The strength of this study is that this CMBA uses the latest technique, SDM-PSI, for the CBMA.^[11,12]^ Compared with previous CBMA methods, such as the old versions of SDM, Activation Likelihood Estimation (ALE), and Multilevel Kernel Density Analysis (MKDA), SDM-PSI makes major improvements, such as applying a standard subject-based permutation test to control the FWER and use of unbiased estimation of effect sizes, random-effects models, Freedman-Lane-based permutations, and TFCE statistics.^[11,12]^ One of the limitations of this study is that the CBMA is based on peak coordinates information, rather on statistical parametric maps, which may bias the results.

VBM is a popular technique to investigate differences in regional GM volume or density over the whole brain at the group-level. However, it has been suggested that many imaging and methodological factors may affect the results, such as imaging acquisition, preprocessing (realignment and segmentation), modulation, model definition, and statistical analysis.^[10,16]^ We then will review all studies included to address these points.

## Author contributions

**Conceptualization:** HaiCun Shi, ZhenYu Dai, PingLei Pan.

**Data curation:** LiQin Sheng, PanWen Zhao, HaiRong Ma.

**Formal analysis:** HaiRong Ma, LiQin Sheng.

**Funding acquisition:** PingLei Pan.

**Investigation:** LiQin Sheng, PanWen Zhao, Liang Qi.

**Methodology:** LiQin Sheng, PingLei Pan.

**Project administration:** LiQin Sheng, PingLei Pan.

**Resources:** PanWen Zhao, YuanYuan Shi.

**Software:** LiQin Sheng.

**Supervision:** HaiCun Shi, ZhenYu Dai, PingLei Pan.

**Validation:** PingLei Pan.

**Visualization:** YuanYuan Shi, LiQin Sheng.

**Writing – original draft:** LiQin Sheng, PanWen Zhao, HaiRong Ma.

**Writing – review & editing:** HaiCun Shi, JianGuo Zhong, ZhenYu Dai, PingLei Pan.
